# GLYCATION-LOWERING COMBO EXTENDS LIFESPAN, IMPROVES INSULIN, REDUCES CALORIC INTAKE

**DOI:** 10.1093/geroni/igad104.3569

**Published:** 2023-12-21

**Authors:** Pankaj Kapahi

**Affiliations:** Buck Institute for Research on Aging, Novato, California, United States

## Abstract

Non-enzymatic reactions in glycolysis lead to the accumulation of methylglyoxal (MGO), a reactive precursor to advanced glycation end-products (AGEs), which has been suggested to drive obesity- and aging-associated pathologies. We observe that a combination of nicotinamide, lipoic acid, thiamine, pyridoxamine and piperine, which were selected to lower glycation (Gly-Low), reduce toxic glycolytic byproducts, MGO and MGO-derived AGE, MG-H1. Administration of Gly-Low reduced food consumption and body weight, improving insulin sensitivity and survival in both leptin receptor deficient (Lepr db) and wildtype C57 control mouse models. Unlike calorie restriction, Gly-Low inhibited ghrelin-mediated hunger responses and upregulated Tor pathway signaling in the hypothalamus. Gly-Low also extended lifespan when administered as a late life intervention, suggesting its potential benefits in ameliorating age-associated decline by inducing voluntary calorie restriction and reducing glycation.

